# A Theoretical Model for ROP Localisation by Auxin in *Arabidopsis* Root Hair Cells

**DOI:** 10.1371/journal.pone.0008337

**Published:** 2009-12-16

**Authors:** Robert J. H. Payne, Claire Suzanne Grierson

**Affiliations:** School of Biological Sciences, University of Bristol, Bristol, United Kingdom; Lund University, Sweden

## Abstract

**Background:**

Local activation of Rho GTPases is important for many functions including cell polarity, morphology, movement, and growth. Although a number of molecules affecting Rho-of-Plants small GTPase (ROP) signalling are known, it remains unclear how ROP activity becomes spatially organised. *Arabidopsis* root hair cells produce patches of ROP at consistent and predictable subcellular locations, where root hair growth subsequently occurs.

**Methodology/Principal Findings:**

We present a mathematical model to show how interaction of the plant hormone auxin with ROPs could spontaneously lead to localised patches of active ROP via a Turing or Turing-like mechanism. Our results suggest that correct positioning of the ROP patch depends on the cell length, low diffusion of active ROP, a gradient in auxin concentration, and ROP levels. Our theory provides a unique explanation linking the molecular biology to the root hair phenotypes of multiple mutants and transgenic lines, including OX-ROP, CA-rop, *aux1*, *axr3*, *tip1*, *eto1*, *etr1*, and the triple mutant *aux1 ein2 gnom* ^eb^.

**Conclusions/Significance:**

We show how interactions between Rho GTPases (in this case ROPs) and regulatory molecules (in this case auxin) could produce characteristic subcellular patterning that subsequently affects cell shape. This has important implications for research on the morphogenesis of plants and other eukaryotes. Our results also illustrate how gradient-regulated Turing systems provide a particularly robust and flexible mechanism for pattern formation.

## Introduction

Rho small GTPases are a large family of highly conserved signalling proteins that contribute to biological processes as diverse as host-pathogen interactions, wound healing, development, and cancer [1], [2]. They play fundamental roles in eukaryotic cell division, cell morphogenesis and cell movement, through effects on actin and microtubule cytoskeletons, gene expression, and enzyme activity. The intracellular location of these proteins is important, and in plants the active forms of certain Rhos accumulate in patches that induce local cell outgrowths. Activation of the Rho-of-Plants (ROPs) proteins may occur by transcriptional up-regulation of ROP expression, or by the modulation of ROP activity via ROP-regulators (including ROP-GEFs and ROP-GAPs) that might themselves be transcriptionally or post-transcriptionally regulated [3], [4]. One of the best systems for studying ROP activity is the developing root hair (RH) cell.

**Figure 1:**
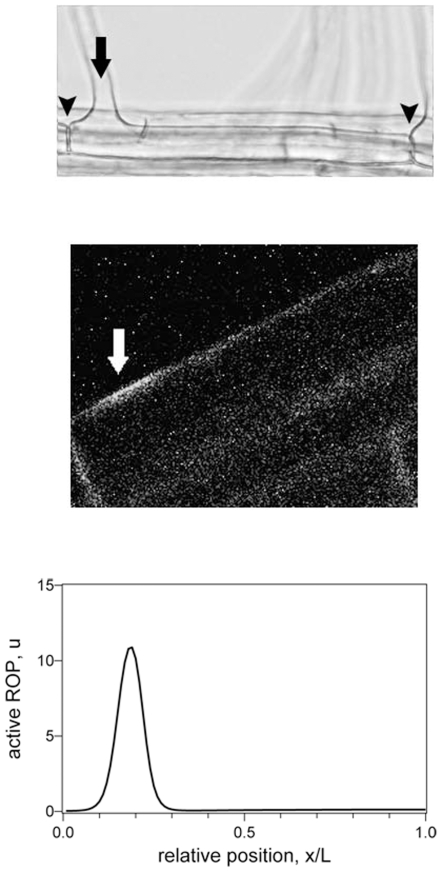
Wildtype Root Hair Formation. Basal cell ends are to the left. **A** Position of root hair on a wildtype root hair cell. Arrow indicates root hair outgrowth, arrowheads denote end walls. **B** Fluorescence image of ROP2-Green Fluorescent Protein in a young root hair cell approximately 45 µm long, showing the site from which a hair will begin to grow within the next few minutes. **C** Simulation plot of concentration of active ROP, *u(x,t)*, against distance for a cell 50 µm long (% distance relative to cell length); a high concentration represents ROP localisation and thus indicates the position where a hair will subsequently develop; see main text for parameters.

RH cells produce hairs that make up the majority of the root surface area of many crops and play an essential role in nutrient and water uptake from the soil, in anchorage, and in interactions with pathogens and symbionts. Development of RHs unfolds in a well-known sequence. RH cells are first formed at the root tip, and subsequently elongate while migrating away from the tip [5]. In many plant species each RH cell has a single hair placed close to the basal end of the cell (end nearest the root tip) [6], [7], an arrangement that leads to regular spacing of root hairs, and is thought to help maximise nutrient uptake [8]. A typical wildtype root hair is shown in Figure 1A and an example of the accumulation of ROPs prior to hair growth in Figure 1B. Type I ROPs accumulate at predictable sites on the RH cell membrane where growth is about to take place, and RH growth is stimulated when ROP activity is experimentally increased [9], [10].

Root hair development is regulated by the plant hormone auxin. Auxin-mediated degradation of AUX/IAA proteins has been identified as affecting RH position, as well as many other aspects of root hair development including initiation, timing, and growth [11], [12], [13], [14], [15], [16]. Experimental manipulations show that auxin influences the site on the cell membrane where patches of ROP form, and hence the site on the cell of root hair outgrowth. Moreover, in a mutant background in which auxin transport is severely disrupted (*aux1 ein2 gnom^eb^*) exogenous auxin induces root hairs to grow at the end of the cell nearest to the auxin source, even when this is the opposite end from normal [16].

Molecular mechanisms of root hair positioning have eluded conventional genetic approaches. It has proved difficult to isolate mutants in which the subcellular location of root hair outgrowth is altered, and difficult to interpret those mutants that do exist, including *rhd6*, *procuste1*, and auxin and ethylene mutants [7], [16], [17]. Here we take an alternative approach, treating RH positioning as an example of biological patterning. Historically, models of biological patterning have considered multicellular patterns. The so-called Turing mechanism has been particularly influential, and has been suggested as a possible source of patterning in diverse developmental processes, such as hair follicle patterning in skin, pigmentation patterning in fish, and skeletal development in limbs [18], [19], [20]. More recently, theoreticians have started to apply the same types of ideas at the level of a single cell. Recent studies suggest that related Rho GTPases from other organisms are well suited to spontaneous pattern formation via a Turing or Turing-like mechanism [21], [22], [23], [24], [25]. Turing patterns are sometimes referred to as diffusion-driven instabilities because a key condition is that the different chemicals diffuse at different rates. In the ROP system the active form of type I ROP is expected to have a lower diffusion coefficient than the inactive form on account of it being tightly associated with the cell membrane via an S-acyl group [26], and so ROPs, like Rhos, are naturally suited to act as Turing morphogens.

Existing studies of pattern formation by Rhos have focussed predominantly on explaining cell polarity. In contrast, the pattern we seek to explain in RH cells is more complex, in that the hair is usually set a little way back from the cell end, and that there exist various mutant phenotypes with multiple hairs [27], [10]. A basic Turing mechanism by itself is not enough to give the patterning of ROP localisation seen in root hair cells. In this paper we hypothesise that the extra factor required is a gradient in either the parameter controlling autocatalysis of ROP activation, or in the rate of production of inactive ROP. The ability of a regulatory gradient to stabilise Turing patterns has been noted before [28], although this important property is rarely mentioned and has been little studied. In light of what is known about the importance of auxin in root hair development, we hypothesise that auxin is the obvious candidate for providing the regulatory gradient. The presence of such a gradient is supported by recent multicellular models [29], [15], which predict an auxin gradient at the level of a cell.

## Results

### The Model

**Figure 2:**
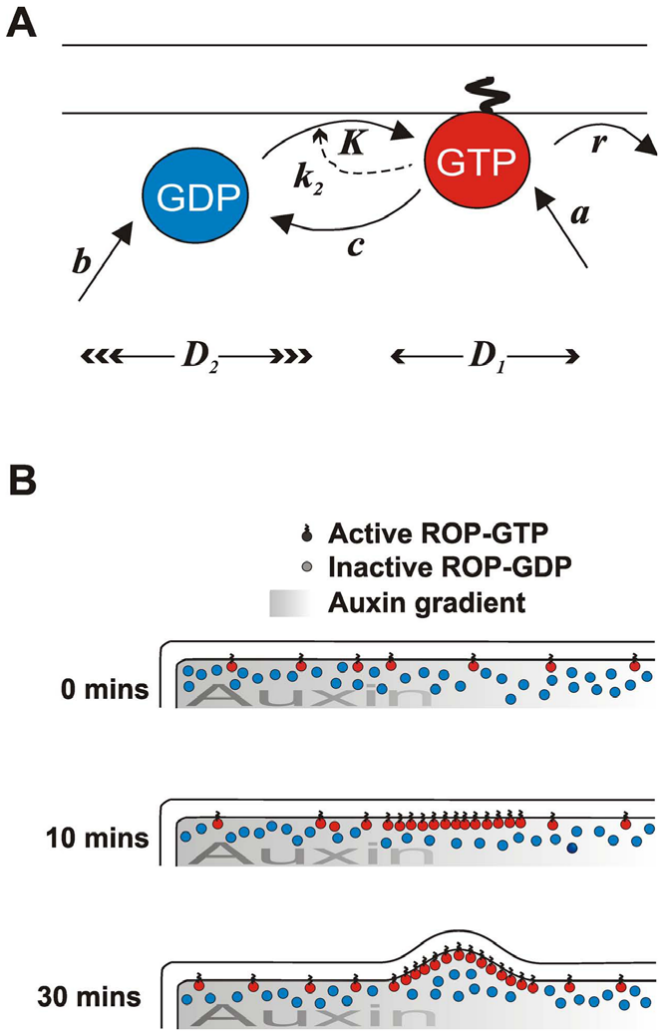
Model Parameters and Interpretation. **A** The model considers two types of Rho of Plants (ROPs) molecules: active, GTP-bound ROPs, represented by variable *u(x,t)*, which are S-acylated, strongly associated with the cell membrane, and diffuse slowly (*D_1_*), and inactive GDP-bound ROPs, represented by variable *v(x,t)*, which are not S-acylated, associate weakly with the membrane or not at all, and diffuse much more quickly (*D_2_*). For the other parameters, see main text. **B** Schematic interpretation of events near the basal end of the cell. At the outset (0 minutes) active and inactive ROPs are assumed randomly distributed around the cell periphery, and active ROPs spend most of their time attached to the membrane via their S-acyl groups. As time passes a ROP patch self-assembles towards the basal end of the cell (10 minutes), causing local root hair growth (30 minutes).

We use a mathematical reaction-diffusion model in which *u*(*x*,*t*) and *v*(*x*,*t*) represent concentrations of a generic active ROP (more strongly bound to the membrane) and a generic inactive ROP (weakly associated with the membrane), respectively. This simple ROP-based Turing system is summarized in Figure 2A and can be represented by two coupled partial differential equations:
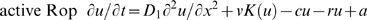
(1)

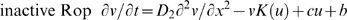
(2)where the function 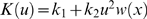 is the rate of ROP activation. ROP activation is assumed to be auto-catalysed by active ROP. Biological mechanisms for ROP autocatalysis have been proposed, for example via scaffold proteins that might recruit and activate ROP GEFs [4]; auto-activation of Rho GTPase through an effect on GEF activity has been modeled in yeast [22]. To mimic an auxin gradient regulating the ROP activation rate we impose a spatial gradient described by *w*(*x*). In this model, unbinding of active ROP occurs at rate *c*, inactive ROP is created at rate *b* and active ROP at rate *a*. Active ROP is removed from the system at rate *r* (for example by degradation, recycling, or some form of irreversible binding). The diffusion coefficients for the active and inactive ROP are *D*_1_ and *D*_2_ respectively, with 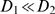. We solve the equations numerically in a one dimensional region 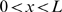, with zero-flux boundary conditions. It is sensible to use only a one dimensional system because this helps us focus on the most important characteristics of root hair positioning, which relate only to the longitudinal axis of the cell.

### Parameter Values

Precise parameter values are not available. Therefore, in order to use estimates that reasonably reflect the known biology, we choose values used for comparable parameters from amongst the various modelling studies of Rhos [21], [22], [23], [25]. Except where otherwise stated, the parameters used are as follows: 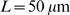, 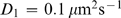, 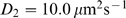, 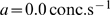, 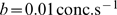, 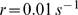, 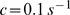, 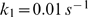, 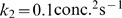 (where conc. is the unit of concentration). For the spatial gradient we use a form which represents an exponential decline across the cell length, 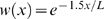. This form was chosen to give a gradient, in terms of the drop of auxin concentration along the length of a cell, of a similar magnitude to that predicted by simulations in Jones *et al.* [15].

The length *L* is not the final cell length, but the cell length at which the patch of ROP is presumed to become fixed in position. We run each simulation for the equivalent of 15 minutes of model time, and allow the model domain (which represents cell length) to elongate by the equivalent of 1 µm per 100 seconds. This growth rate is consistent with measurements in Sugimoto *et al.* [30], and the duration of the simulation is comparable to the time from appearance of a patch of ROP to the start of swelling (a more exact comparison is not possible because the appropriate initial conditions are not known; we use homogeneous initial conditions for *u*(*x*,*t*) and *v*(*x*,*t*)). It is assumed that thereafter there is no change to the relative position of a patch regardless of the actual final cell length (Fischer *et al.*, 2006). In the output of the simulations, a peak in concentration of *u*(*x*,*t*) represents a peak in concentration of active ROP, and we interpret this as indicating a site of hair growth (Figure 2B).

The term *k*_2_ *w*(*x*) implicitly accounts for the hypothesised action of auxin. The biological mechanism of the action is not yet known, but a rational choice for the parameter value can be made based on an understanding of the mathematics of Turing patterns. For an homogenous domain (no spatial gradient in parameters) it is possible to mathematically derive a complete set of criteria for instability leading to patterning [18], [19]. The analysis predicts there will usually either be no pattern, or a set of regularly spaced peaks of concentration across the whole cell length, depending on parameter values. When the parameters are such that a pattern may form, they are said to be inside the ‘Turing space’ (an abstract construct within a multi-dimensional parameter-space) [31]. To obtain the wildtype pattern we therefore choose *k*_2_ *w*(*x*) such that the parameters lie within the homogeneous Turing space at the basal end of the cell, but are beyond or near the edge of the Turing space towards the apical end.

In the model just described, it is assumed that the auxin acts to impose a gradient on the parameter *k_2_*. It is easy to modify the model to test alternative hypotheses for auxin action. This is done by changing the position of *w*(*x*) within the equations. For example, to test the hypothesis that the auxin acts to modify the rate of creation of inactive ROP, one would impose a gradient on the parameter *b* to give the form *bw*(*x*). We used this method to test a range of hypotheses for the way that the auxin might regulate the ROP kinetics.

### The Wildtype Phenotype

**Figure 3:**
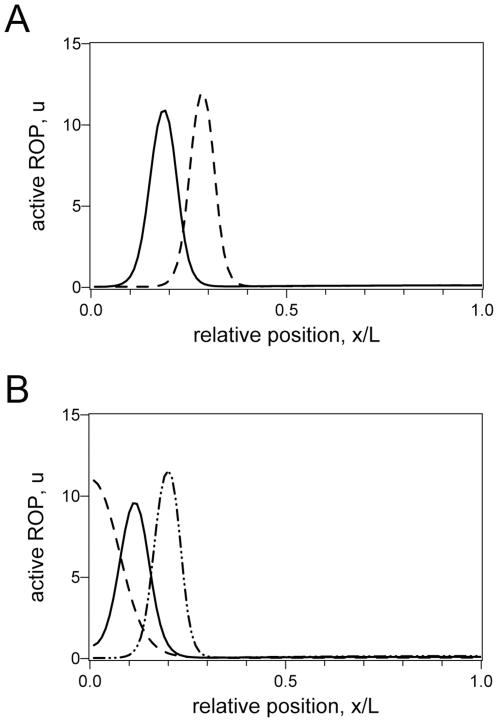
The Effects of Timing and Cell Length. In all figures the basal cell end is to the left. All parameters are as for wildtype, except as noted. **A** If the time until the bulge appears is slower than wild type then the hair is shifted apically: simulation plot assuming time until bulge forms is 15 minutes (solid line) versus 30 minutes (dashed line). **B** Cell length at time of hair initiation shifts the final relative hair position: Solid line *L* = 40 µm; shorter cell shifts towards basal end (dashed line, *L* = 20 µm); longer cell shifts towards the apical end (dash-dot line, *L* = 60 µm).

In a wildtype root hair cell the ROPs become localised in a patch positioned towards the basal end of the cell (Figure 1B). This pattern can readily be produced by our model, as illustrated in Figure 1C. In our simulations the patch first forms at the extreme basal end of the cell and then moves in an apical direction, gradually slowing until an equilbirum position is reached. The formation of the bulge that leads to hair growth is not explicitly included in our model, but happens on a time scale shorter than that for the patch of localised ROP to reach a spatial equilibrium. Fig 3A illustrates how the position of the hair is affected by the length of time until the patch transforms into a bulge. Below we discuss how the model can mimic the phenotypes of various genetic mutants and transgenic lines, as summarised in Table 1.

**Table 1:** Mutant genotypes and phenotypes discussed in the Results.

Genotype	Figure	Relevant phenotype	Biological mechanism	[Ref]	Modelling
wildtype	(1C)	hair near basal end	—	—	default parameters
*aux1 ein2 gnom^eb^*		variable position, disrupted auxin synthesis and transport	flattened auxin gradient	[16]	flat *w*(*x*) with perturbations
OX-ROP	(4C)	multiple hairs	more production of unbound ROP	[10]	larger *b*
CA-rop		multiple hairs	more delivery of bound ROP	[10]	larger *a*
*tip1*	(4D)	basal shift, wide, short	Active ROP less sticky (?), shorter cell	[40], [27]	larger *D*_1_, lower *L*
*aux1*	(4A)	apical shift	reduced auxin transport	[51], [15]	larger *k*_2_
*aux1*	(4B)	multiple hairs	reduced auxin transport	[51]	even larger *k*_2_
*axr3*		bald	less response to auxin	[33]	smaller *k*_2_
*eto1*	(3B)	basal shift	ethylene over-expression, short cell	[7]	smaller *L* (?)
*etr1*	(3B)	apical shift	ethylene over-responsive, long cell	[7]	larger *L* (?)

Items marked (?) are more speculative.

### Auxin Profile and Cell Lengthening

It is not possible to measure auxin gradients within cells, but it is possible to experimentally disrupt auxin synthesis and transport, as in the triple mutant *aux1 ein2 gnom^eb^*. In experiments by Fischer *et al.* [16] this triple mutant was found to have a hair placed at various places in the cell, including some towards the wrong (i.e. apical) end of the cells. In our simulations the hair can be placed anywhere in the cell by changing the profile of the auxin distribution, and the hair can be reliably positioned using auxin profiles with as little as 10% difference between the maximum and minimum values of *w(x)*. Thus our model predicts the distribution of hair positions that would be expected if auxin transport were disrupted with an auxin profile that is roughly flat but with some degree of fluctuation, as is believed to be the case in *aux1 ein2 gnom^eb^*.

Our simulations indicate that in qualitative terms the results are remarkably robust against the details of the gradient function. In particular, in the apical region the profile of *w(x)* is of almost no consequence.

The effect of cell length is more subtle. It is well known that domain length has an important role in determining the pattern in homogeneous Turing systems [19], [32]. Domain length is similarly influential in our heterogeneous context. In essence, changing the cell length alters the relative balance between diffusive processes and kinetic processes, and so changes the bounds of the Turing space. In simulations we find that when the cell length is shorter than wildtype the peak is shifted towards the basal end, whereas if the cell length is longer the peak is shifted towards the apical end (Figure 3B). In our simulations the parameter *L* reflects the cell length at the time when the hair is first initiated, not the final length, and so in general we predict that mutants or growth conditions in which initiation occurs earlier (thus on a shorter cell) will produce a basal shift in hair position. There are no known mutants whose only action is to alter cell length at the time of root hair initiation, although see the discussions of the *tip1*, *eto1* and *etr1* mutants, below.

Since the cell length is important, it stands to reason that cell lengthening should also be important. In practice we find that the rate of cell growth is slow enough as to not have a large effect on the outcome of the simulations.

### Auxin Mutants

**Figure 4:**
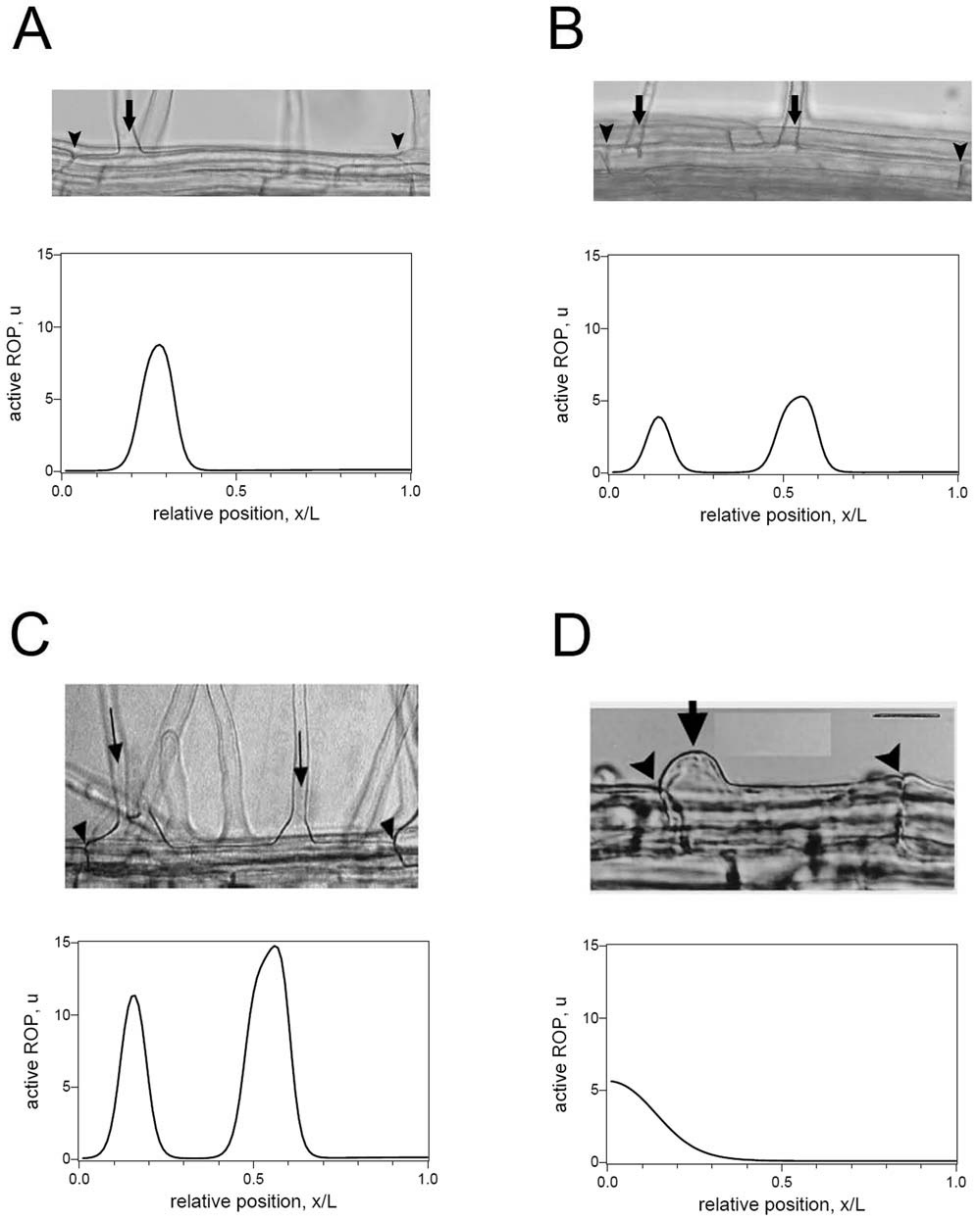
Examples of Auxin and ROP mutants. In all figures the basal cell end is to the left. Each panel shows an actual root hair and, beneath, a simulation output. All parameters are as for wildtype, except as noted. **A** Auxin mutant *aux1*-7, *k*_2_ = 0.2 conc.^2^s^−1^. **B** Auxin mutant *aux1*-22, *k*_2_ = 0.8 conc.^2^s^−1^. **C** ROP mutant OX-ROP, *b* = 0.03 conc.s^−1^. **D** ROP mutant *tip1*, *D*_1_ = 0.5 µm^2^s^−1^, *L* = 30 µm.

Phenotypes of *aux1* mutants require careful interpretation. Mutations in *AUX1* are known to reduce auxin transport into cells, but it is not intuitively obvious how this might affect auxin levels in the zone of hair initiation. Simulations in Jones *et al.* [15] predict that, given an adequate auxin supply from the root tip, reduced AUX1 activity could lead to an increase of auxin in the zone in which the root hair is initiated. Thus we mimic the effect of this mutant by increasing *k*_2_, the parameter that represents ROP activation in response to auxin. Specifically, *k*_2_ controls the degree to which active ROP autocatalyses its own activation. Figures 4A and 4B show the model output when using 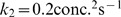 and 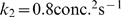, which exhibit an apical shift in hair position and a double haired phenotype, respectively. These compare well with examples of observed phenotypes of *aux1* mutants (Figure 4).

In the *axr3* mutant the degradation of the AXR3 protein in response to auxin is reduced at least 7-fold [33], delaying the auxin response, and the RH cells remain hairless [12]. In our model the action of this mutant can be mimicked by reducing *k*_2_ 7–10 fold. The output after 15 minutes matches the observed phenotype, with the simulations showing no localisation of active ROP, and hence no local outgrowth.

Mutations in the *ETO1* (ethylene overproduction) and *ETR1* (ethylene resistant, lacking the ETR1 ethylene receptor) genes both affect the hair phenotype, showing a basal shift and an apical shift, respectively [7]. Ethylene up regulates production of auxin in the root apex [34], [35], but it is not known whether the *eto1* and *etr1* mutants have altered levels of cellular auxin in cells that are initiating hairs. It is known, though, that these mutants modify the cell length, causing shorter and longer cells, respectively [36], [37], [38]. We therefore model these mutants just by altering *L*. Using values of *L* = 20 µm and *L* = 60 µm in the model results in shifts similar to the direction of measured shifts in hair position of *eto1* and *etr1*, respectively [7] (c.f. Figure 3B).

### Altering ROP Activity

Both the OX-ROP and the Constitutively Active (CA)-rop transgenic lines are capable of producing cells with two hairs. The OX-ROP lines used by Jones *et al.* [10] express about twice as much ROP gene product as wildtype plants, effectively doubling the pool of inactive ROP available for activation. CA-rop transgenic lines produce similar levels of ROP to OX-ROP, but point mutations have been introduced into the ROP amino acid sequence so that the CA-rop is synthesised in its active form [10]. We therefore represent these mutants by setting 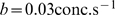 for OX-ROP, and 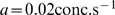 for CA-rop. The resultant phenotype is similar in both cases. Figure 4C shows an example for OX-ROP.

Active ROP is believed to be S-acylated [26], which is expected to increase the strength of anchorage of active ROP to the cell membrane by a factor of approximately 2 to 5 fold [39]. TIP1 is an S-acyl transferase that we speculate could potentially S-acylate active ROPs in root hairs. Experiments to test this possibility are in progress in the Grierson laboratory. The *tip1* mutant has shortened cell length [40], and shows a phenotype in which the base of the hair is wider than normal and is shifted basally [27]. To explore what would happen if active ROP was not S-acylated, we mimicked the *tip1* mutant by increasing the effective diffusion coefficient of active ROP to 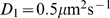, as well as using a shorter cell length *L* = 30 µm. The model output compares well with the observed phenotype, as illustrated in Figure 4D.

### Alternative Hypotheses

So far we have presented results under the assumption that the auxin gradient modifies the rate of autocatalysis by the activated ROP (i.e. the auxin affects the parameter *k*_2_). Other model variants were tested, representing alternative hypotheses about the action of the auxin gradient. Of these other model variants several were capable of producing the required wildtype pattern, but only one other was also capable of mimicking the range of phenotypes of the mutants and transgenic lines. This variant represents the hypothesis that the auxin acts to modify the rate of creation of inactive ROPs (i.e. the auxin affects the parameter *b*). The results under this hypothesis were qualitatively similar to those already presented (Figures 3, 4), and so we do not repeat them here.

### Other Mutants

Phenotypes that resemble other mutants, such as *scn1* [27], [41], *procuste* [17], and weak *gnom* alleles [16], can also be obtained from the model, but we do not present them here because the biological mechanisms for these mutants are not well enough understood to be confidently interpreted. For example, although confocal microscopy suggests that *scn1* mutants have an unusually high proportion of ROP attached to the membrane [41], it is not known what proportion of the membrane-attached ROP is active. Although we can produce *scn1*-like phenotypes with our model in a variety of ways, it is not clear how to represent *scn1* in the terms of the model. We have restricted our results to cases where there is a good enough understanding of the biological mechanisms involved. Interpretation of mutants in which there is both earlier initiation (thus decreased cell length at initiation, predicted to move the hair basally), and increased auxin levels (predicted to move the hair apically) will be particularly problematic.

## Discussion

Our *in silico* experiments shed light on the possible role of auxin in the development of root hairs. Although the mechanisms by which auxin influences ROP activity are not known, our results are strongly supportive of the hypothesis that a cellular auxin gradient upregulates the net amount of active ROP. The kinetics of ROPs are less well characterised than those of Rhos, and so our model is necessarily kept simple. It is a strength of the model that, despite this, it is able to mimic the ROP localisation patterns of such a range of root hair mutants and transgenic lines. In doing so, our model provides a valuable bridge between the genetics, molecular biology, and mutant phenotypes of root hair morphogenesis.

We are not the first to model the dynamics of patterning in individual cells. The role of Rhos in cell polarity has now been modelled for a number of cell types, including in yeast and *Dictyostelium*, and for migrating neutrophils [21], [22], [23]. When looking at Rho cycling in motile eukaryotic cells, Mori *et al.* [25] used a wave-pinning model to explain cell polarity. They argued that a Turing mechanism is not a viable process at the level of a cell because it is unable to produce patterns fast enough. Our situation is somewhat different, in that the speed at which patches of ROP appear on RH cells need not occur at anything like the speed at which motile eukaryotic cells need to respond to signals. In our simulations the concentration of active ROP typically approached a suitable distribution in about 10–15 minutes, which is in keeping with the observed time for a pre-hair swelling to appear [42].

Our model is able to produce more sophisticated patterns than the simple polarity produced by previous studies of patterning in single cells. This is partially because RH cells are typically longer than the eukaryotic cells previously modelled: mathematical theory tells us that changing domain length is equivalent to changing the ratio of diffusion coefficients to kinetic coefficients [19]. In other words we expect different cell lengths to produce different phenotypes, even when all other parameters are unchanged. The importance of cell length in determining the outcome of morphogenesis in our model echoes the recent realisation that signalling outcomes can be altered by changing cell length and shape [43], [24]. Root hair biologists do not usually collect cell length data at the time of root hair initiation, but we recommend that such measurements should be made.

It is interesting to note that the results show various ways in which the hair position can be shifted apically. We also observe that for any parameter change which shifts hair position apically, a further change in the parameter can cause a double haired phenotype. We therefore predict that the proportion of double hairs is likely to correlate with the amount of apical shift.

Our study also emphasizes the importance of post-translational modifications, such as S-acylation, which alter the diffusivity of proteins. Mathematical theory tells us that the ratio of diffusion coefficients is central in determining the form of patterning in Turing systems [19]. In root hair cells, the inactive and active states of ROPs are good candidates for possible Turing morphogens on account that their diffusivities are likely to be strongly affected by post-translational modifications, especially the S-acylation of active ROP [26]. Protein-protein interactions, such as those between inactive ROP and ROP GDI [41], or between active ROPs and membrane- or cytoskeleton-associated proteins, are also likely to alter ROP mobility, and hence regulate the localization of patches. Thus the diffusion ratio of active and inactive ROPs is likely to strongly affect the root hair phenotype, and this may be an interesting avenue for future experimental work.

Whilst active and inactive ROPs can self-organise into patches, it is only when a spatial gradient is imposed on one of the model parameters that phenotypes comparable to root hair cells are exhibited. To date there has been little formal mathematical theory on the role of heterogeneous domains on Turing patterns [44], [45]. The idea of controlling a Turing pattern with an imposed gradient was suggested 20 years ago for stripe formation during Drosophila development [28]. The theory was criticised at the time for not matching the understanding of gap-gene proteins in Drosophila segmentation, although a contribution by Turing-like mechanisms was not ruled out [46]. The biology of Rhos is very different from that of Drosophila gap genes. Understanding of our model is helped by an appreciation of the mathematics of Turing systems in an homogeneous domain, and in particular the role of the Turing space [31]. The wildtype ROP localisation becomes possible when the auxin gradient is such that at the basal end of the cell the parameter values fall deeper within the Turing space than at the apical end.

For the simulations shown in the figures we assumed the auxin gradient to act as a modifier of the rate of autocatalysis of active ROP (gradient acts on *k*_2_). But very similar plots were also attained using an assumption that the auxin acts to modify the rate of introduction of inactive ROPs (gradient acts on *b*). Although our results illustrate the importance of the gradient, existing modeling and experimental results do not allow us to distinguish between these two hypotheses. There are precedents in the literature for autoactivation [4], [22], but it remains plausible that ROPs could be actively transported [4]. These possibilities, together with the roles and relative significance of various ROPs and ROP regulators, will have to be tested experimentally.

*Arabidopsis* has several advantages for the necessary experimental tests. Because patches of active ROP produce irreversible changes in the shapes of plant cell walls, ROP activation leaves a permanent record from which it is relatively easy to collect data, and root hair ROPs can be observed in very young cells on living plants, so it is possible to monitor the entire process of patch formation. This contrasts with many other model cells, where Rho-related structures are transient, difficult to observe, or cannot be studied *in vivo*. It should be possible to use established procedures to obtain a range of data, including RH cell lengths, intracellular auxin distributions [29], [15], and ROP diffusion coefficients, the latter using fluorescence recovery after photobleaching (FRAP) or photoactivatable or photoconvertible fluorescent proteins. It should also be possible to observe ROP dynamics in *tip1* mutants and, using existing point-mutated ROPs that cannot be S-acylated, test our hypotheses about the mechanism of TIP1 action. Another important feature is the extent to which known Arabidopsis mutants are available as a means of verifying the model. Remarkably few Turing models have been compared with phenotypes from more than one genotype. Miura *et al.* [47] used a mixed-mode Turing pattern to explain the Doublefoot mutant mouse limb, and Turing models of the development of Arabidopsis trichomes on the leaf blade were recently tested against a suite of mutants and transgenic lines [48], [49]. Mathematical models have also been compared with mutants to explain the multi-cellular arrangement of Arabidopsis root hair and non hair cells [50].

Our theory explains how interaction of the plant hormone auxin with ROPs might spontaneously lead to localised patches of active ROP. Our results suggest that correct positioning of the ROP patch depends on the cell length at the time of patch formation, the relatively low diffusion of active ROP compared to inactive ROP, a gradient in auxin concentration, and overall levels of inactive ROP in the root hair cell. Given the recent experimental evidence for the role of auxin in RH positioning [16], simulation evidence for an intracellular auxin gradient [29], [15], and comparative evidence from dynamic models of polarization of Rhos [21], [22], [23], [24], [25], our predictions merit rigorous experimental testing. The root hair system offers an excellent opportunity to investigate how dynamic molecular interactions generate cell morphology.

## Materials and Methods

### Plant Material and Growth Conditions

Columbia, OX-ROP, CA-rop, *aux1-7*, *aux1-22*, *axr3*, *tip1* have been described elsewhere [10], [16], [12], [27].

For phenotyping, plants were grown on solid growth medium [27] for 5 days. For laser confocal microscopy and measurement of root-hair elongation rate, seeds were grown for 5 days beneath a thin film of solid growth medium on a glass coverslip and sealed in a humid chamber. All plants were grown under a regime of 16 h light, 8 h dark.

### Microscopy

Slides were mounted onto the microscope stage of a Leica DM IRBE microscope and digital images collected using a Leica DFC 350 FX camera. Fluorescent images were collected as described previously [10].

### Simulations

Model simulations were run using XPP (v5.96) available from G. Bard Ermentrout at http://www.math.pitt.edu/~bard/xpp/xpp.html.
